# Supplementary material for: Antenatal physical exercise level and its associated factors among pregnant women in Hawassa city, Sidama Region, Ethiopia
Source: PLoS One. 2023 Apr 28;18(4):e0280220. doi: 10.1371/journal.pone.0280220 (PMC10146453; doi:10.1371/journal.pone.0280220)
Supplement: S1 Appendix — (DOCX) [file pone.0280220.s001.docx]

# Annex

**Hawassa University**

**College of Medicine and Health Science**

**Department of Clinical Midwifery**

## Annex I: Consent Form

Hello! My name is …………….I am currently a member of data collector in this Study that assess women’s knowledge, practice, and associated factors on physical exercise during pregnancy among pregnant women attends antenatal care for an investigator doing his research at Hawassa university, south Ethiopia. He has received permission from Hawassa University College of medicine and health Science ethical issue committee. Furthermore, he has got permission latter from Hawassa city administrator health department office. You were selected for the study by hoping that you will cooperate with me. I am kindly requesting you to answer the questions that I have prepared for you. You will not be provided any incentives or payment to take part in this research. I assure you all information gathered during the study will be kept completely confidential. All the information that you are going to deliver to me will be coded for anonymity. Only the principal investigator will have access to the data.

You have a full right to refuse part or the whole questionnaires & no one enforces you to do so. However, your honest participation and answers to the questionnaire will help us in a better understanding of the problem and give guidance on how to intervene in the study area. So do you be willing to participate in the study? 1. Yes…… 2. No……..

Having been well explained and informed of the intentions and benefits of the Study I voluntarily consent to participate in the study. (*If yes, let her sign and go ahead, if No stop here*.).

Respondents` signature …………… Date…………….

Annex -2: Assent form

My name is -------------------------I am asking you to take part in research study because of we are trying to learn more about women’s knowledge, practice, and associated factors on physical exercise during pregnancy among pregnant women attends antenatal care for an investigator doing his research at Hawassa university, south Ethiopia.. If you agree to be in this study since your child or sisters age is less than 18 years old and this age is not allowed in Ethiopia constitution to give information required. So you are kindly requested to give information from your child’s point of view or you are expected to more understand about the research and final agree that she can give information .I need you to provide me your honest answer to the questions you want to respond as this would help us to come up with genuine conclusions and recommendations that would be used as evidence. Participating in this research project will not have direct benefit to you also there is no risk that comes to as pregnant women. So, make sure that, there should be no harm caused because you are involved in this study. You have right not to start or terminate it if something unclear happen.

. I have understood what will be you doing for this study

All my questions have had answered

I have talked to your parents and agreed to take part in this research.

Do you agree to give the required information? Yes No

Or parents agree Yes No

## Annex –3 Participant information sheet

You have been chosen to participate in this study Knowledge, Practice and associated Factors on physical exercise among Pregnant Women attend antenatal care at the health facility of Hawassa city administration. Please listen me the following statements, and if you have any unclear questions you can ask before you agree to participate.

1. Topic: Women’s knowledge, practice and its associated factors on physical exercise during pregnancy in Hawassa city, Sidama Regional State, Southern Ethiopia, 2021.

2. Objective of the study: The objective of this study is to assess women’s knowledge, Practice and its associated Factors on physical exercise during pregnancy among Pregnant Women attend antenatal care at health facility Hawassa city administration, Hawassa, Ethiopia 2021. The information you provide will be helpful to Hawassa city administration health office, health care providers and office authorities, the communities and government to provide plan and strategy on pregnancy and maternal health-related programs.

3. Participation Procedure and guidelines; the information you provide will be kept completely anonymous. Your name will not be written on the form. Your answers are completely confidential. The questions are prepared in English, and Amharic language as a structured interviewer administered questionnaire. It will take about 20-25 minutes to complete the interview.

4. Participant benefit and risk; your participation in the study does not involve risks. You might feel some mild discomfort from responding to some items on the questionnaires, but the risk of discomfort is not greater than other normal activities you have in the home. You also may experience some benefits from participating in this study.

5. Right to refuse or withdraw.

a. You do not have to answer any question that you do not want to answer.

b. You may end participation in the study at any time you want, without losing any of your right

6. Persons to Contact: this research project will be reviewed and approved by the Ethical clearance committee of the Department of Midwifery, Hawassa University. If in case you want to know more information about the research and its undertakings, you can contact the committee through the address of the advisors or the principal investigator below.

Dereje Zeleke (BSc in Midwifery). Email: [derejezeleke35@gmail.com](mailto:derejezeleke35@gmail.com)

Zemenu Yohannes (MSc, Assistant professor ). Email:zemenu2013@gmail.com

Teshome Melese (MSc Assistant professor). Email: [teshemele@gmail.com](mailto:teshemele@gmail.com)

Gossa Fetene (BSc MSc in Clinical Midwifery). Email:Feteneg2119@gmail.com

## Annex –3 English version Questioners

Data collection tool study on women’s knowledge, Practice on Physical Exercise during pregnancy and Its Associated Factors among pregnant women attends antenatal care at health facility Hawassa city administration, south, Ethiopia 2021

Name of data collector --------------------------- Signature------------------ Date------------- Name of supervisor------------------------------------------- Signature ------------------- Date----

| Note. The inclusion criteria if “NO” to the first and “Yes” to the second, and thread question, stop the collection of the data and go to the next candidates  1. Does your pregnancy was confirmed? 1. Yes 2. No  2. Have you been interviewed by me on this issue before a time? 1. Yes 2. No  3. Have you been medically advised to minimize physical exercise by health care providers? 1. Yes 2. No | | | | | |
| --- | --- | --- | --- | --- | --- |
| Part 1: socio demographic characteristics | | | | | |
| S.No | Question | | Answer/response | | |
| 101 | How old are you? | | ----------years old | | |
| 102 | What is your Marital Status? | | | 1. Single 2.Married 3. Divorced 4.Windowed 5. Cohabitating | |
| 103 | What is your educational level? | | | 1. Non-formal education 2. Reading and writing 3. Elementary4. High School 5. College or university | |
| 104 | What is your husband educational level? | | | 1. Non-formal education 2. Reading and writing 3. Elementary4. High School 5. College or university | |
| 105 | What is your occupational status? | | | 1. House-wife 2. Farmer 3. Daily laborer  4. Private employer 5. Merchant 6. Governmental employer 7. Other specify………. | |
| 106 | What is your husband occupational status? | | | 1. Farmer 2. Daily laborer  3. Private employer 4. Merchant 5. Governmental employer 6. Other specify | |
| 107 | How Mach is your total monthly income? | | | In Ethiopian birr…….. | |
| Part 2. Pregnancy, and obstetrics related characteristics | | | | | |
| S.NO | | Question | | | Answer/response |
| 201 | | Gestational age from you last menstrual date | | | --------weak |
| 202 | | How many antenatal care visit does you have including the current visit | | | ----------In number |
| 203 | | What is your pregnancy status? | | | 1 planned 2 unplanned |
| 204 | | How many times have you got pregnancy, including the current pregnancy? | | | ----in number |
| 205 | | How many children do you have? | | | ---------------in number |
| 206 | | Have you had abortion previously? | | | 1 yes 2 no ( if no Skip to QN 301) |
| 207 | | How many times do you have abortion? | | | ----in number |

Part3. Question related to Knowledge of physical exercise

| S. no | Questions | True | | | False | |
| --- | --- | --- | --- | --- | --- | --- |
| 301 | Physical exercises are important during pregnancy? |  | | |  | |
| 302 | Breathing physical exercise is essential during pregnancy? |  | | |  | |
| 303 | Walking physical exercise is necessary during pregnancy? |  | | |  | |
| 304 | Dancing physical exercise is important during pregnancy? |  | | |  | |
| 305 | Running physical exercise is important during pregnancy? |  | | |  | |
| 306 | Cycle driving physical exercise is essential during pregnancy? |  | | |  | |
| 307 | Pelvic floor physical exercise is essential during pregnancy? |  | | |  | |
| 308 | Ankle and toe physical exercise is necessary during pregnancy? |  | | |  | |
| Questions related to benefits of physical exercise during pregnancy for mothers | | | | Answerer | | |
|  |  |  |  | True | | False |
| 309 | Physical exercise during pregnant has important for the mothers? | | |  | |  |
| 310 | Physical exercise during pregnant can shorten duration of labor? | | |  | |  |
| 311 | Physical exercise during pregnant can reduce fatigue and stress? | | |  | |  |
| 312 | Physical exercise during pregnant can reduce depression? | | |  | |  |
| 313 | Physical exercise during pregnant can reduce risk of preeclampsia? | | |  | |  |
| 314 | Physical exercise during pregnant can reduce risk of diabetes mellitus? | | |  | |  |
| 315 | Physical exercise during pregnant can reduce the chance of cesarean sections? | | |  | |  |
| Questions related to benefits of physical exercise during pregnancy for fetus | | | Answerer | | | |
|  |  |  | True | | False | |
| 316 | Physical exercise during pregnant has important to the fetus? | |  | |  | |
| 317 | Physical exercise during pregnant reduce risk of preterm birth? | |  | |  | |
| 318 | Physical exercise during pregnant reduces risk of abortion? | |  | |  | |
| 319 | Physical exercise during pregnant reduces risk of fetal macrosomia? | |  | |  | |
| 320 | Physical exercise during pregnant reduces risk of low birth weight? | |  | |  | |
| 321 | Physical exercise during pregnant reduce fetal distress during labor? | |  | |  | |
| Questions related to contraindications of physical exercise during | | | Answer | | | |
|  |  |  | True | | False | |
| 322 | Exercise in supine position is safe during pregnancy period? | |  | |  | |
| 323 | Heavy weightlifting is safe during pregnancy period? | |  | |  | |
| 324 | Exercises result in marked straining is safe during pregnancy period? | |  | |  | |
| 325 | Exercise in dehydration condition is safe during pregnancy period? | |  | |  | |
| 326 | Exhausting tennis match is safe during pregnancy period? | |  | |  | |
| 327 | Long distance running is safe during pregnancy period? | |  | |  | |
| Questions related to resumption of physical exercise during pregnancy | | | | Answer | | |
|  |  |  |  | True | | False |
| 328 | If vaginal bleeding is occurring during physical exercise, resumption of it is essential? | | |  | |  |
| 329 | If abdominal pain happens or painful contractions is started during physical exercise, resumption of it is necessary? | | |  | |  |
| 330 | If amniotic fluid is leaked during physical exercise, resumption of it is mandatory? | | |  | |  |
| 331 | If headache is happened during physical exercise, resumption of it is best? | | |  | |  |
| 332 | If chest pain is occurring during physical exercise, resumption of it is best measure? | | |  | |  |
| 333 | If muscle weakness that affecting body balance is occurs during physical exercise, continuing it will have risks? | | |  | |  |
| 334 | If calf pain or body swelling is occurs during physical exercise, continuation of it is not recommended? | | |  | |  |

Part 4. Question about health service related characteristics

| No. | Questions | | Answer | |
| --- | --- | --- | --- | --- |
|  |  |  | Yes | No |
| 401 | Is there any facility which supports you to do physical exercise? | |  |  |
| 402 | Is there sport field in your surroundings to do physical exercise? | |  |  |
| 403 | Have you been advised by health professional to perform physical exercise prior to the current pregnancy? | |  |  |
| 404 | Do you follow mass media | |  |  |
| 405 | From who/where you have got information about physical exercise? | 1. Health professional, 2. Mass media, 3. Family/friend, 4. Book, 5.Other specify ----- | | |

Part5. Question related to practice towards physical exercise during pregnancy

| s.no | Question | Answer | | |
| --- | --- | --- | --- | --- |
| 501 | Did you practice exercise before current pregnancy | 1. Yes 2. No | | |
| 502 | Did you practice exercise during current pregnancy? | 1. Yes 2. No | | |
|  | | No at all | Yes daily for <30 mints or weekly for < 3 days/ <150 mints | Yes daily ≥30 mints or weekly for ≥3 days/ ≥150mints |
| 503 | Did you have practiced walking exercise? |  |  |  |
| 504 | Did you have practiced dancing exercise? |  |  |  |
| 505 | Did you have practiced cycle drivingexercise? |  |  |  |
| 506 | Did you have practiced breathing exercise? |  |  |  |
| 507 | Did you have practiced pelvic floor exercises? |  |  |  |
| 508 | Did you have practiced ankle and toe exercise? |  |  |  |
