# Supplementary material for: Antenatal physical exercise level and its associated factors among pregnant women in Hawassa city, Sidama Region, Ethiopia
Source: PLoS One. 2023 Apr 28;18(4):e0280220. doi: 10.1371/journal.pone.0280220 (PMC10146453; doi:10.1371/journal.pone.0280220)
Supplement: S2 Appendix — (DOCX) [file pone.0280220.s002.docx]

የመረጃ ወረቀት
በሃዋሳ ዩኒቨርሲቲ
የህክምና እና የጤና ሳይንስ ኮሌጅ
ሚዴዋይፈሪ ትምህርት ክፍል

## አባሪ I: የስምምነት ቅጽ

እንደምን አደሩ/አረፈዱ/ዋሉ፡፡ ስሜ ------------------- እባላለው ፡፡ እኔ በአሁኑ ስአት የቅድመ ወሊድ እንክብካቤ የሚከታተሉ ነፍሰ ጡር ሴቶች ላይ በእርግዝና ወቅት ስለ አካል ብቃት እንቅስቃሴ በተመለከተ ያላቸዉን እውቀት፣ ልምምድ እና ተዛማጅ ጉዳዮች ለማወቅ የድህረ-ምረቃ ተማሪ በሆኑት አቶ ደረጀ ዘለቀ በሚያደርጉት ጥናት ዉስጥ የመረጃ ሰብሳቢ አባል ነኝ ፡፡ ጥናቱን ለመሰራት ከሐዋሳ ዩኒቨርሲቲ የህክምና እና የጤና ሳይንስ ሥነምግባር ጉዳይ ኮሚቴ ፈቃድ አግንቷል ፡፡. እንዲሁም በትጨማሪ ከሃዋሳ ከተማ አስተዳዳር ጤና መምሪያ ጽ / ቤት ህጋዊ ፈቃድ አግንቷል ፡፡. ከእኔ ጋር ይተባበራሉ በሚል ተስፋ ለጥናቱ በእጣ ተመርጠዋል ፡፡ የተዘጋጁትን ጥያቄዎች እንዲመልሱ በትህትና እጠይቃለሁ ፡፡. በዚህ ጥናት ውስጥ ለመሳተፍ ምንም ዓይነት ማበረታቻ ወይም ክፍያ አይሰጥዎትም ፡፡. በጥናቱ ወቅት የተሰበሰቡ ሁሉም መረጃዎች ሙሉ በሙሉ በሚሰጥር የሚያዙ መሆናቸዉን አረጋግጥልወታለው ፡፡ የእርስዎን ማንነት በሚስጥር ለመጠበቅ ሲባል ስምዎት በመጠይቅ ቅጹ ላይ አይጻፍም ፡፡ እንድሁም መረጃውን ማግኘት የሚችሉት ዋና መርማሪ እና የምርምር ረዳቶች ብቻ ናቸው።.

በጥናቱ ለመሳተፍ ፈቃደኛ ነዎት?? 1. አዎ. . . 2. አይደለሁም( አመሰግናለው ብለው ይሰናበቱቸው)

የጥናቱን ዓላማዎች እና ጥቅሞች በደንብ ከተረዳሁ በኋላ በጥናቱ ለመሳተፍ ፈቃደኛ ነኝ ፡፡.

የምላሽ ሰጪ ፊርማ ... . . . . . . ቀን. . . . .

## አባሪ -2 የተሳታፊ መረጃ ወረቀት።

በሀዋሳ ከተማ አስተዳድር ጤና ተቋም ውስጥ የቅድመ ወሊድ እንክብካቤ በሚከታተሉ ነፍሰ ጡር ሴቶች ላይ በእርግዝና ወቅት ስለ አካል ብቃት እንቅስቃሴ በተመለከተ ስላላቸዉ እውቀት፣ ልምምድ እና ተዛማጅ ጉዳዮች ጥናት ውስጥ እንዲሳተፉ ተመርጠዋል ፡፡ እባክዎን የሚከተሉትን መረጃወች አነብልወታለሁ ማንኛውንም ግልጽ ያልሆነልወትን ጥያቄዎችን መጠየቅ ይችላሉ ፡፡.

1. ርዕስ-በሃዋሳ ከተማ ፣ በሲዳማ ክልል ፣ በደቡባዊ ኢትዮጵያ ፣ በእርግዝና ወቅት የአካል ብቃት እንቅስቃሴን በተመለከተ የሴቶችን ዕውቀት ፣ ልምምድ እና ተዛማጅ ሁኔታዎችን በተመለከተ አጠቃላይ ጥናት ይሆናል ፡፡.

2. የጥናቱ ዓላማ እና ጥቅም:-የዚህ ጥናት ዓላማ በሀዋሳ ከተማ አስተዳድር ጤና ተቋም ውስጥ የቅድመ ወሊድ እንክብካቤ በሚከታተሉ ነፍሰ ጡር ሴቶችን በእርግዝና ወቅት ስለአካል ብቃት እንቅስቃሴ ያላቸውን ዕውቀት ፣ ልምምድ እና ተዛማጅ ጉዳዮች ለማወቅ ነው ፡፡ የሚሰጡት መረጃ በእርግዝና እና ከእናቶች ጤና ጋር የተዛመዱ መርሃግብሮችን እና እቅዶችን ለማቅረብ ለሀዋሳ ከተማ አስተዳደር የጤና መምሪያ ጽ/ቤት ፣ ለጤና ባለሙያ እና ባለሥልጣናት ፣ ለማህበረሰቡ እና ለመንግስት ጠቃሚ ይሆናል ፡፡

3. የተሳትፎ ሥነ ሥርዓት እና መመሪያዎች፡- የሚሰጡት መረጃ ሙሉ በሙሉ ማንነቱ ሳይታወቅ ይቀመጣል ፡፡ በቅጹ ላይ ስምዎ አይፃፍም። መልሰዎ ሙሉ በሙሉ ሚስጥራዊ ይሆናሉ። ጥያቄዎቹ በእንግሊዝኛ ፣ በሲዳሚኛ እና በአማርኛ ቋንቋ የተዘጋጁ ቃለ-መጠይቅ ናቸው ፡፡. ቃለመጠይቁን ለማጠናቀቅ 25 ደቂቃ ያህል ሊወስድብወ ይቸላል ፡፡

4. የተሳታፊ ጥቅም እና ጉዳት፡- እርስዎ በዚህ ጥናት ሊይ በመሳተፍዎ የተነሳ ምንም አይነት ጉዳት አይደርስብወትም።. ለአንዳንድ ጥያቄወች ምላሽ በሚሰጡበት ጊዜ ትንሽ የመረበሽ ስሜት ሊሰማዎት ይችላል ፣ እንዲሁም በዚህ ጥናት ውስጥ በመሳተፍወ አንዳንድ ጠቃሚ መረጃወችን ሊያገኙ ይችላሉ ፡፡.

5. ያለመስማማት ወይም መጠይቁን የማቐረጥ መብት።.ሀ. መልስ መስጠት የማይፈልጉትን ማንኛውንም ጥያቄ አለመመለስ ይችላሉ ፡፡. ለ. ሙሉ ቃለመጠይቁ መጨረስ ካልፈለጉ በማንኛውም ጊዜ የማቐረጥ መብት አለወት ፡፡

6. መረጃ የሚያገኙባቸው ሰዎች-ይህ የምርምር ጥናት የሚገመገመው በሃዋሳ ዩነበርስቲ የህክምና እና ጤና ሳይንስ ኮሌጅ ሥነ ምግባር ማረጋገጫ ኮሚቴ ነው ፡፡. ስለ ጥናቱ እና ስለ ሥራው የበለጠ መረጃ ማወቅ ከፈለጉ ፣ ኮሚቴውን ከዚህ በታች ባለው አድራሻ በኩል ማግኘት ይችላሉ ፡፡.

ዋና መርማሪ- ደረጀ ዘለቀ (BSc). አድራሻ: derejezeleke35 @ gmail.com

ዋና አማካሪ - - ዘመኑ ዮሃንስ (ረዳት ፕሮፌሰር). አድራሻ:- zemenu2013 @ gmail.com

ተባባሪ አማካሪ- ተሸመ መለሰ ((ረዳት ፕሮፌሰር)) አድራሻ: teshemele@gmail.com

## አባሪ -3 ጥያቂዎች።

በደቡባዊ ኢትዮጵያ፣ ሲዳማ ክልል፣ ሀዋሳ ከተማ አሰተዳድር ባሉ ጤና ተቋም ውስጥ የቅዴመ ወሉድ እንክብካቤ በሚከታተሉ ነፍሰ ጡር ሴቶች ላይ በእርግዝና ወቅት ስለ አካል ብቃት እንቅስቃሴ በተመለከተ ስላላቸዉ እውቀት፣ ልምምድ እና ተዛማጅ ጉዳዮች የመረጃ መሰብሰቢያ ቅጽ፡፡

የመረጃ ሰብሳቢው ስም - ------------------ ፊርማ--------------------------------

የተቆጣጣሪው ስም---------------------------ፊርማ--------------መጠይቅ ኮድ . . . . . . .

| ማስታወሻ. ቃለመጠይቁን ለመጀመር ቅድመ ሁኔታወች:- ለጥያቄ 1 መልስ “አይደለሁም”፣ ለለጥያቄ 2 እና 3 መልስ “አዎ”፣ ከሆነ መረጃ መሰብሰቡን ያቁሙና ወደ ቀጣዩ እጩዎች ይሂዱ።.  1. እርግዝናወ የተረጋገጠ ነው? 1. አዎ 2. አይደለሁም  2. በዚህ በፊት በዚህ ርዕሰ ጉዳይ ላይ ቃለ-መጠይቅ ድርጌወታለው ? 1. አዎ 2. አይደለሁም  3. የአካል እንቅስቃሴን እንዲቀንሱ ወይም የእረፍት እንድወሰዱ በሕክምና ሰራተኞች ተመክረዋል? አዎ 2. አልተባልኩም | | | | |
| --- | --- | --- | --- | --- |
| ክፍል 1-ማህበራዊ እና ስነ ህዝባዊባዊ ህሪያት፡፡ | | | | |
| ተቁ | ጥያቄ | | የጥያቄ መልስ / ምላሽ። | |
| 101 | ዕድሜዎ ሰንተነው?? | | - ------ ዓመት | |
| 102 | የጋብቻ ሁኔታዎ ምንድን ነው? | | 1ያላገባች-- 2.ያገባች------------3.የተለያየች---------4.ባልየሞተባት------- 5. ሳይጋቡ አብራ የምትኖር----- | |
| 103 | የትምህርት ደረጃዎ ምንድን ነው? | | 1.ያልተማረች- 2.ማንበብና መጻፍ የምትችል 3.አንደኛ ደረጃ ያጠናቀቀች 4.ሁለተኛ ደረጃ ያጠናቀቀች 5.ኮሌጅ ወይም ዩኒቨርሲቲ የገባቸ | |
| 104 | የባልወ ትምህርት ደረጃ ምንድን ነው? | | 1.ያልተማረ- 2.ማንበብና መጻፍ የሚችል 2.አንደኛደረጃ ያጠናቀቀ 3.ሁለተኛ ደረጃ ያጠናቀቀ 4.ኮሌጅ ወይም ዩኒቨርሲቲ የገባ | |
| 105 | የሥራ ሁኔታዎ ምንድን ነው | | 1. የቤት-እመቤት 2. ገበሬ 3. የቀን ሰራተኛ።4. የግል ተቀጣሪ 5. ነጋዴ 6. የመንግስት ሰራተኛ 7. ሌላ ካለ ይግለጹ፡ | |
| 106 | የባልወ ሥራ ሁኔታ ምንድን ነው | | 1. ገበሬ 2. የቀን ሰራተኛ 3. የግል ተቀጣሪ 4. ነጋዴ 5. የመንግስት ሰራተኛ 6. ሌላ ካለ ይግለጹ፡ | |
| 107 | አጠቃላይ ወርሀዊ የገቢ መጠንወ ስንት ነው | | ...................የኢትዮጵያብር | |
| ክፍል 2 እርግዝና እና ከእናቶች ጋር የተዛመዱ ባህሪዎች። | | | | |
| S.No | | ጥያቄ | | የጥያቄመልስ / ምላሽ። |
| 201 | | የአሁኑ እርግዝና ስንት ወር ሁኖታል? | | - - - - ወር። |
| 202 | | የአሁኑን ጨምሮ ለምን ያክል ጊሄ የቅድመ ወሊድ ክትትል አድርገዋል | |  |
| 203 | | የእረግዝናው ሁኔታ ምንድን ነው | | 1 የታቀደ 2 ያልታቀደ |
| 204 | | የአሁኑን እርግዝና ጨምሮ ስንት ጊዜ አርግዘው ያውቃሉ? | | በቁጥር |
| 205 | | ስንት ልጆች አሉዎት? | | በቁጥር |
| 206 | | በእርግዝና ጊዜ ዉርጃ አጋጥሞወት ያውቃል? | | 1. አዎ 2. አያውቅም (አያውቅም ከሆነ ወደ ጥ.ተ.ቁ. 301ዝለል) |
| 207 | | ስንት ጊዜ አስወርዶወት ያዉቃል? | | በቁጥር |

ጥያቄክፍል3።ነብሰጡር እናቶች በእርግዝና ወቅት ስለ አካልብቃት እንቅስቃሴ ያላቸውን እውቀት በተመለከተ

| ተ.ቁ | ጥያቄዎች | | መልስ | | | |
| --- | --- | --- | --- | --- | --- | --- |
|  |  |  | እውነት | | ሀሰት | |
| 301 | ቅድመ ወሊድ የአካል ብቃት እንቅስቃሴ በእርግዝና ጊዜ ጠቃሚ ነው? | |  | |  | |
| 302 | በመተንፋፈስ የሚሰራ የአካልብቃትእንቅስቃሴ በእርግዝና ጊዜ ጠቃሚ ነው? | |  | |  | |
| 303 | የእግርጉዞ( ወክ) አካልብቃት እንቅስቃሴ በእርግዝና ጊዜ ማድረግ ጠቃሚ ነው? | |  | |  | |
| 304 | የዳንስ አካልብቃት እንቅስቃሴ በእርግዝና ጊዜ ማድረግ ጠቃሚ ነው ? | |  | |  | |
| 305 | የሩጫ አካልብቃት እንቅስቃሴ በእርግዝና ጊዜ ማድረግ ጠቃሚ ነው??? | |  | |  | |
| 306 | በእርግዝና ጊዜ ሳይክል በመንዳት አካልብቃት እንቅስቃሴ ማድረግ ጠቃሚ ነው? | |  | |  | |
| 307 | በእርግዝና ጊዜ የዳሌ ወለል አካልብቃት እንቅስቃሴ ማድረግ ጠቃሚ ነው?? | |  | |  | |
| 308 | በእርግዝና ጊዜ የቁርጭምጭሚት እና የእግርጣቶች አካልብቃት እንቅስቃሴ ማድረግ ጠቃሚ ነው? | |  | |  | |
| በእርግዝና ወቅት የአካል ብቃት እንቅስቃሴ መስራት ለእናቶች ያለውን ጠቀሜታ በተመለከተ | | | | መልስ | | |
|  |  |  |  | እውነት | ሀሰት | |
| 309 | በእርግዝና ወቅት የአካል ብቃት እንቅስቃሴ መስራት ለእናቶች ጤና ይጠቅማል | | |  |  | |
| 310 | በእርግዝና ወቅት የአካል ብቃት እንቅስቃሴ መስራት የምጥ መርዘምን ይቀንሳል | | |  |  | |
| 311 | በእርግዝና ወቅት የአካል ብቃት እንቅስቃሴ መስራት ድካም እና ጭንቀት ይቀንሳል | | |  |  | |
| 312 | በእርግዝና ወቅት የአካል ብቃት እንቅስቃሴ መስራት ድብርትን ይቀንሳል | | |  |  | |
| 313 | በእርግዝና ወቅት የአካል ብቃት እንቅስቃሴ መስራት በእርግዝና ምክንያት የሚከሰትን ከፍተኛ የደም ግፊት ይከላከላል | | |  |  | |
| 314 | በእርግዝና ወቅት የአካል እንቅስቃሴ መስራት በእርግዝና ምክንያት የሚከሰት የስኳር በሽታን ይከላከላል/ይቀንሳል | | |  |  | |
| 315 | በእርግዝና ወቅት የአካል ብቃት እንቅስቃሴ መስራት ቀዶጥገና የመሰራት እድልን ይቀንሳል | | |  |  | |
| በእርግዝና ወቅት የአካል ብቃት እንቅስቃሴ መስራት ለፅንሱ ያለው ጠቀሜታ በተመለከተ ጥያቄወች | | | | መልስ | | |
|  |  |  |  | እውነት | | ሀሰት |
| 316 | በእርግዝና ወቅት የአካል ብቃት እንቅስቃሴ መስራት ለፅንሱ ጠቀሜታ አለው | | |  | |  |
| 317 | በእርግዝና ወቅት የአካል ብቃት እንቅስቃሴ መስራት ጊዛው ሳይደርስ ምጥ እንዳይጀምር ይከላከላል | | |  | |  |
| 318 | በእርግዝና ወቅት የአካል ብቃት እንቅስቃሴ መስራት የፅንስ መውረድ አደጋን ይቀንሳል | | |  | |  |
| 319 | በእርግዝና ወቅት የአካል ብቃት እንቅስቃሴ መስራት የፅንስ ከመጠን በላይ ማደግ አደጋ እንዳይከሰት ይከላከላል | | |  | |  |
| 320 | በእርግዝና ወቅት የአካል ብቃት እንቅስቃሴ መስራት የፅንስ ከመጠን በታቸ ማነስ አደጋ እንዳይከሰት ይከላከላል | | |  | |  |
| 321 | በእርግዝና ወቅት የአካል ብቃት እንቅስቃሴ መስራት በምጥ ጊዜ የፅንስ መታፈንን እነዳይከሰት ይከላከላል | | |  | |  |
| በእግዝና ወቅት የአካል ብቃት እንቅስቃሴ መስራት የሚከለከልባቸው ጉዳዮች በተመለከተ ጥያቄ | | | | መልስ | | |
|  |  |  |  | እውነት | ሀሰት | |
| 322 | | በእርግዝና ወቅት በጀርባ ተኝቶ የአካል ብቃት እንቅስቃሴ መስራት ጉዳት የለውም | |  |  | |
| 323 | | በእርግዝና ወቅት ከባድ ክብደት በማንሳት የአካል ብቃት እንቅስቃሴ መስራት ጉዳት የለውም | |  |  | |
| 324 | | በእርግዝና ወቅት በጣም ድካም የሚያስከትሉ የአካል ብቃት እንቅስቃሴ መስራት ጉዳት የለውም | |  |  | |
| 325 | | በእርግዝና ወቅት የሰውነት ድርቀት በሚከሰትብተ ስአት የአካል ብቃት እንቅስቃሴ መስራት ጉዳት የለውም | |  |  | |
| 326 | | በእርግዝና ወቅት በጣም አድካሚ የሆነ የቴኒስ ግጥሚያ የአካል ብቃት እንቅስቃሴ መስራት ጉዳት የለውም | |  |  | |
| 327 | | በእርግዝና ወቅት ረጅም ርቀት በሮጥ የአካል ብቃት እንቅስቃሴ መስራት ጉዳት የለውም | |  |  | |
| በእርግዝና ወቅት የአካል ብቃት እንቅስቃሴ መስራት የሚቐረጥባቸው ጊዜያቶች በተመለከተ ጥያቄ | | | | መልስ | | |
|  |  |  |  | እውነት | ሀሰት | |
| 328 | | የአካል ብቃት እንቅስቃሴ በሚሰሩበት ጊዜ ከማህፀን ደም የሚፍሰስ ከሆነ እንቅስቃሴ ማቆም አለበን | |  |  | |
| 329 | | የአካል ብቃት እንቅስቃሴ በሚሰሩበት ጊዜ የምጥ ሕመም ወይም የሆድ ቁርጠት ሰሜት የሚኖር ከሆነ ማቆም ጠቃሚ ነው | |  |  | |
| 330 | | የአካል ብቃት እንቅስቃሴ በሚሰሩበት ጊዜ የእንሽርት ዉሀ የሚፍሰ ከሆነ እንቅስቃሴውን ማቆም ጥሩ ውሳኔ ነው | |  |  | |
| 331 | | የአካል ብቃት እንቅስቃሴ በሚሰሩበት ጊዜ የእራስ ምታት ህመም ስሜት የሚኖር ከሆነ እንቅስቃሴውን ማቆም አስፈላጊ ነው | |  |  | |
| 334 | | የአካል ብቃት እንቅስቃሴ በሚሰሩበት ጊዜ የሰውነት እብጠት እና የታፋ ህመም ሲከሰት ከሆነ የአካል ብቃት እንቅስቃሴውን መቀጠል አይመከርም | |  |  | |
| ጥያቄ ክፍል 5 ነብሰጡር እናቶች በእርግዝና ወቅት መስራተ ስላለባቸው የአካል ብቃት እንቅስቃሴ በተመለከተ ጥያቄ | | | | | | |

| ክፍል 4. ስለ ጤና አገልግሎት ተዛማጅ ባህሪዎች በተመለከተ ጥያቄ | | |  |  |  |
| --- | --- | --- | --- | --- | --- |
| ተ.ቁ | ጥያቄወች | | | መልስ | |
|  |  |  |  | አወ | የለም |
| 401 | የአካል ብቃት እንቅስቃሴ ለማድረግ የሚረዳዎ ተቋም አለ? | | |  |  |
| 402 | የአካል ብቃት እንቅስቃሴ ለማድረግ በአካባቢያችሁ ተስማሚ የሆነ ቦታ አለ? | | |  |  |
| 403 | ከማርዝወ በፊት የአካል ብቃት እንቅስቃሴ እንዲያደርጉ በጤና ሰራትኛ ተመክረዋል? | | |  |  |
| 404 | ብዙሃን መገናኛ ዜዴወችን ይጠቀማሉ? | | |  |  |
| 405 | ስለ አካል ብቃት እንቅስቃሴ በተመለከተ መረጃ ከማን / ከየት ነው ያገኙት? | 1. ከጤና ሰራተኛ 2. ከብዙሃን መገናኛ፣ 3. ከቤተሰብ / ከጓደኛ፣ 4. ከመጽሐፍ፣ 5. ሌላ ካለ ይግለጹ --- | | | |
|  | | | | | |

| 501 | ከማርገዝወ በፊት የአካል ብቃት እንቅስቃሴ ይሰሩ ነበር? | | 1. አዎ 2. አልሰራም | | | |
| --- | --- | --- | --- | --- | --- | --- |
| 502 | በአሁኑ እርግዝና ወቅት የአካል ብቃት እንቅስቃሴ ልምምድ ያደርጋሉ? | | | 1. አዎ 2. አልሰራም | |  |
|  |  | ሰርቸ አላዉቅም | | አዎ በየቀኑ <30 ደቂቃ ወይም በየሳምንቱ <3 ቀን | አዎ በየቀኑ ≥30 ደቂቃ ወይም በየሳምንቱ ≥ 3 ቀን | |
| 503 | የእገር ጉዞ(ወክ) የአካል ብቃት እንቅስቃሴ ይሰሪሉ? |  | |  |  | |
| 504 | በመደነስ የአካል ብቃት እንቅስቃሴ ይሰሪሉ? |  | |  |  | |
| 505 | ሳይክል በመንዳት የአካል ብቃት እንቅስቃሴ ይሰሪሉ? |  | |  |  | |
| 506 | በመተንፈስ የአካል ብቃት እንቅስቃሴ ይሰሪሉ? |  | |  |  | |
| 507 | የዳሌ ወለል አካል ብቃት እንቅስቃሴ ይሰሪሉ? |  | |  |  | |
| 508 | የጉልበትና የእግር ጣቶችን አካል ብካት እንቀስቃሴ ይሰሪሉ? |  | |  |  | |
