# Supplementary figures and images for: Antenatal physical exercise level and its associated factors among pregnant women in Hawassa city, Sidama Region, Ethiopia
Source: PLoS One. 2023 Apr 28;18(4):e0280220. doi: 10.1371/journal.pone.0280220 (PMC10146453; doi:10.1371/journal.pone.0280220)

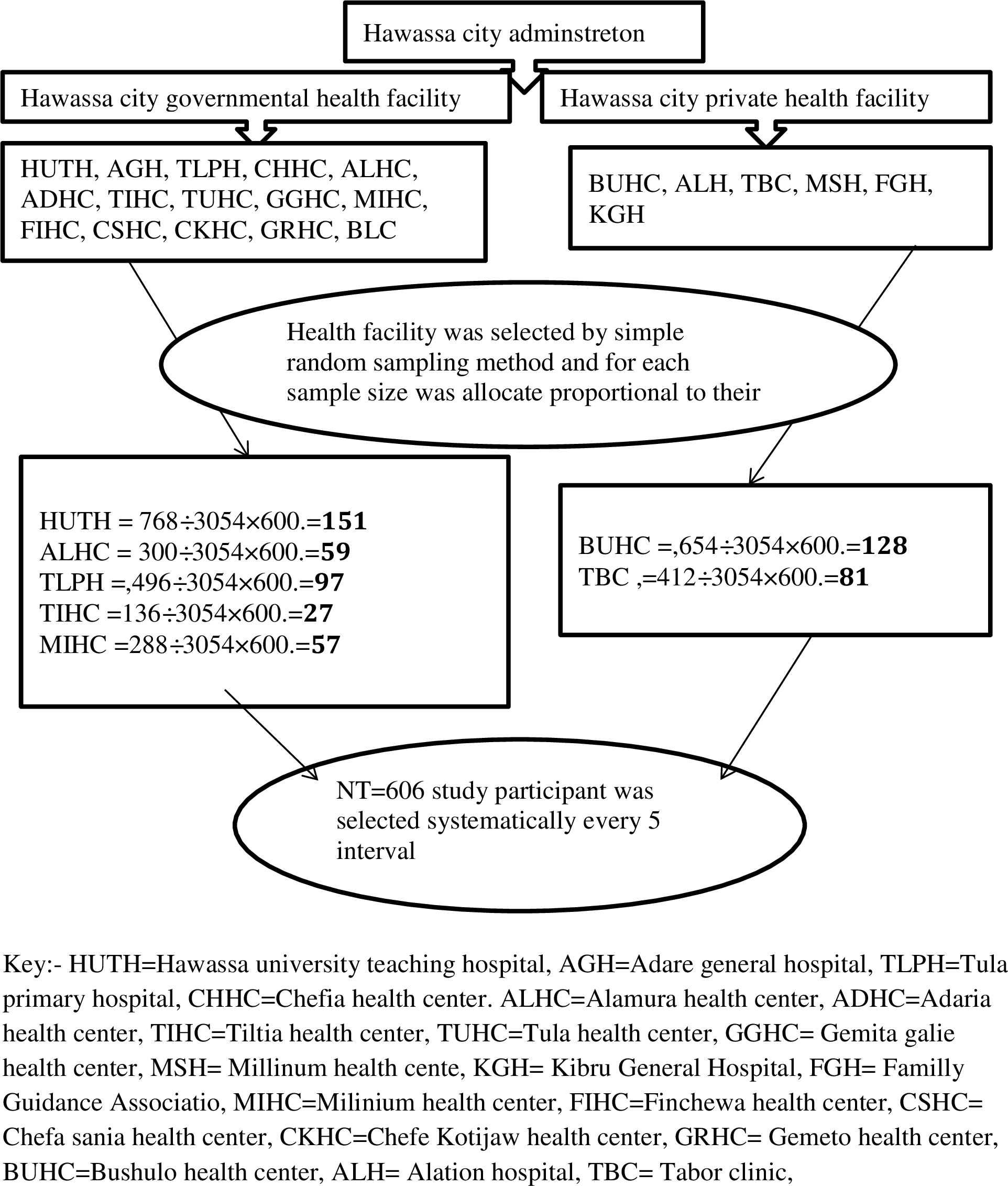

Supplement: S1 Fig — (TIF) [file pone.0280220.s004.tif]

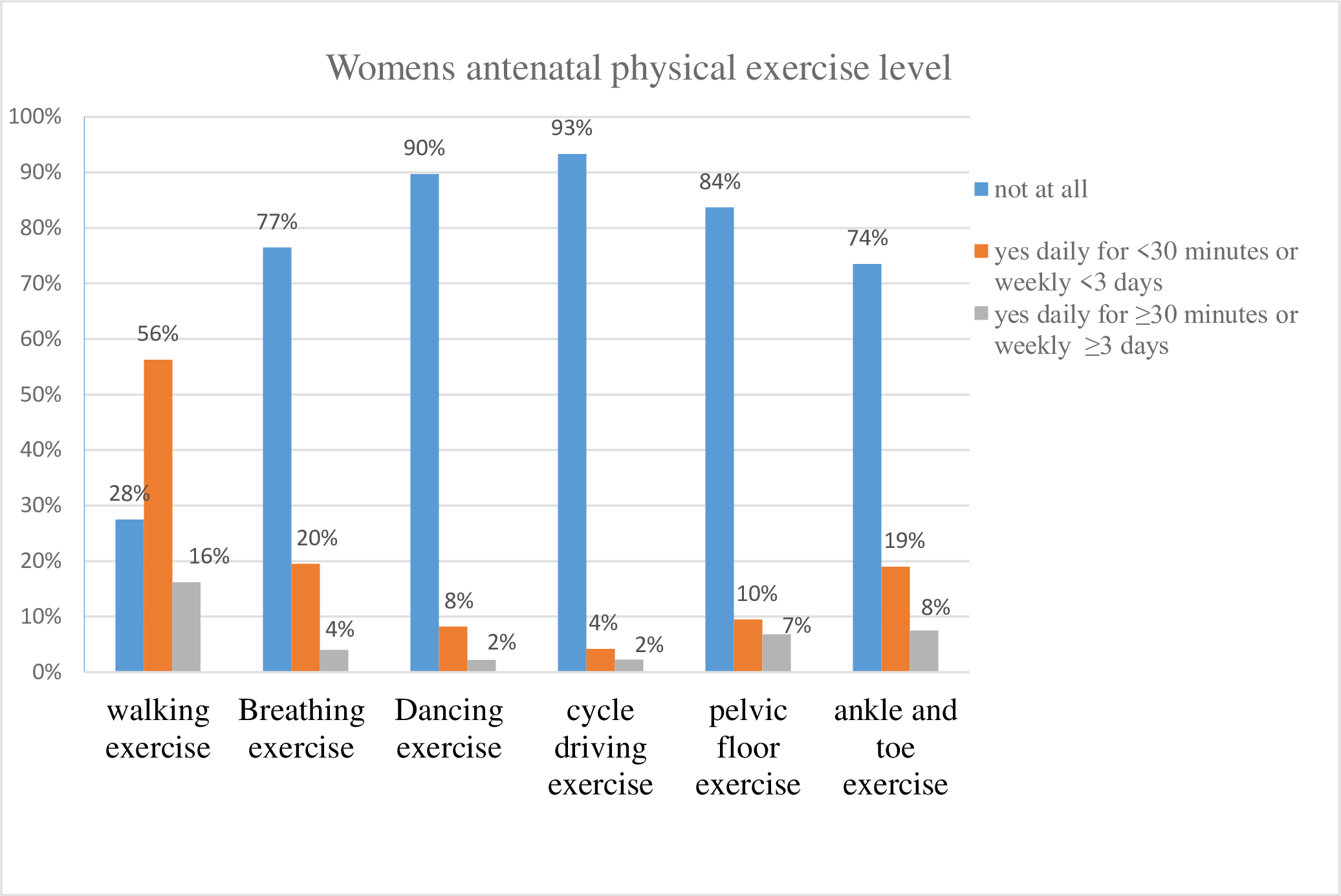

Supplement: S2 Fig — (TIF) [file pone.0280220.s005.tif]
