# Supplementary material for: Antenatal physical exercise level and its associated factors among pregnant women in Hawassa city, Sidama Region, Ethiopia
Source: PLoS One. 2023 Apr 28;18(4):e0280220. doi: 10.1371/journal.pone.0280220 (PMC10146453; doi:10.1371/journal.pone.0280220)
Supplement: Attachment — Submitted filename: Comments to author.docx [file pone.0280220.s006.docx]

**Review comments**

I found this article very interesting aiming to describe physical exercise during pregnancy. However, the authors should change the title to “Physical exercise and its associated factors among pregnant women in Hawassa city, Southern Ethiopia.”

 Abstract

**# Background**

The background is too long and shortens it. Antenatal physical exercise ….what does it mean, the health care providers can order the women during ANC visits? It needs clarity.

**#Result**
There is a punctuation error and you should correct it.

Partners college and above educational level (AOR 1.57, 95% CI: 0.40, 6.12) is not significant. The authors should remove it.

**#Introduction**

The authors should follow the PLoS one citation style.

Maternal Mortality Rate (MMR) remains a global health problem. It can be changed that maternal mortality remains a global health problem.

A study shows that physical exercise during pregnancy in Iran was (39)?

**# Methods**

Did the authors use any materials during data collection, if not authors should remove the material in the methods part.

Your study units are your study populations.  Therefore, the authors should change the population and study unit.

Exclusion

 How to identify critically ill. Did the authors have any checklist to identify critically ill women?

The questionnaire was pretested on 5% (30) of the sample at Adaria general hospital. Where is the city/town? How to control information contamination?

 The authors should clearly state the data collectors’ work area.

**#Results**

It lacks clarity……. majorities (77.3%) of them were in the age group of 20-34 years .

privet employer  spelling error

The author should change the income to IMF income classification way.

\# Discussion

On the hand, women having partners who had college and above education level had 1.7 times higher odds of having an adequate level of practice on antenatal physical exercise during pregnancy compared with those who had no formal education. Check your table this variable is not significant.

Limitations

The authors remove the cross-study as a limitation.

Recommendations

Your recommendation is too long and shortened based on your pertinent findings in your study area.
